# A Double-Layer Dual-Polarized Huygens Metasurface and Its Meta-Lens Antenna Applications

**DOI:** 10.3390/mi14061139

**Published:** 2023-05-28

**Authors:** Shuo Cao, Jianhe Zhou, Ruxue Li, Chunhua Xue

**Affiliations:** Guangxi Colleges and Universities Key Laboratory of Microwave Communication and Micro-Nano Photoelectric Technology, School of Electronic Engineering, Guangxi University of Science and Technology, Liuzhou 545006, China; shuocao2022@163.com (S.C.); liruxueic@163.com (R.L.); xue@gxust.edu.cn (C.X.)

**Keywords:** Huygens’ metasurface, meta-lens, dual-polarized

## Abstract

In this paper, a dual-polarized Huygens unit is proposed, which has a double-layer metallic pattern etched on both sides of one dielectric substrate. Induced magnetism enables the structure to support Huygens’ resonance, thus obtaining nearly complete available transmission phase coverage. By optimizing the structural parameters, a better transmission performance can be achieved. When the Huygens metasurface was used for the design of a meta-lens, good radiation performance was exhibited, with a maximum gain of 31.15 dBi at 28 GHz, an aperture efficiency of 42.7% and a 3 dB gain bandwidth of 26.4 GHz to 30 GHz (12.86%). Due to its excellent radiation performance and very simple fabrication, this Huygens meta-lens has important applications in millimeter-wave communication systems.

## 1. Introduction

Since they were proposed in 2011, metasurfaces have received widespread attention from researchers due to their particular abilities to control electromagnetic (EM) waves [1]. Various physical phenomena have been studied, and related engineering applications, especially antenna applications, have been explored [2,3,4,5,6,7,8,9]. Metasurfaces are usually classified into transmissive and reflective types. Reflective metasurfaces are very easy to create, simply using single-layer metallic patterns as reflection phase shifters to manipulate the phase of the reflected waves on the metal ground plate. In contrast, it is much more difficult to create transmissive metasurfaces using single- or double-layer metallic patterns because of the requirement to simultaneously achieve a high transmission amplitude and 360° transmission phase control [10]. Recently, several approaches of double-layer transmissive metasurfaces have been explored. One approach is the Pancharatnam–Berry metasurface based on the geometric phase. This is mainly used for the manipulation of circular polarized waves [11,12]. Another approach is the transmissive metasurface composed of meandered metallic lines. This is used for polarization conversion [13,14].

In addition, to achieve the ultimate manipulation ability of linear polarized waves, the concept of Huygens’ metasurface (HMS) was introduced in 2013 [15]. Its ultimate manipulation ability of transmitted waves is achieved by managing the coupling between the electrical and magnetic dipoles on the surface. HMS has exhibited interesting physical phenomena such as beam bending [16], airy beams [17], broadband beam manipulation [18], holographic imaging [19,20,21,22,23] and potential antenna applications [24,25]. Due to the easy implementation of electric dipoles, magnetic dipoles are the key to achieving Huygens’ resonance. The methods for implementing magnetic dipoles include vertical metallic ring resonators [15,18] and multilayer cascaded surface unit structures [17,19]. However, such designs make the structure very complicated and difficult to fabricate. Recent investigations show that the magnetic current can be induced by the antisymmetric electric dipole pair, and then the balance between the induced magnetic current and intrinsic electric current can stimulate Huygens’ resonance [26]. By utilizing Huygens’ resonance, HMS can achieve over 400° available transmission phase coverage with double-layer metallic patterns. Such a design greatly simplifies the structural complexity of the transmissive metasurface and provides a more convenient way to manipulate transmitted waves.

The double-layer Huygens metasurface has crucial applications in planar lens antennas. By utilizing the discrete microstrip patches as phase shifters, planar lenses can convert the spherical phase front of an EM wave to a planar phase front based on the optical focusing principle. Then, high-directional radiation can be achieved. Planar lenses are designed based on the multilayer frequency-selective surface method [27,28,29,30], which increases fabrication and assembly costs and thus limits their engineering applications. Compared with a multilayer planar lens, a double-layer Huygens meta-lens can be implemented on only one dielectric substrate and thus has the merits of being easy to fabricate and low-cost. Very recently, double-layer Huygens meta-lens antennas with different functions were reported, including single-polarization [26], dual-polarization [31], bi-functional reflectarray/transmitarray [32] and ultralow profile antennas [33]. The double-layer Huygens metasurface provides new opportunities for achieving lighter planar lens antennas that are easier to manufacture.

In this paper, based on the induced magnetism, a new type of double-layer HMS is proposed. The Huygens unit consists of a cross pattern on the top layer and a split Jerusalem cross pattern on the bottom layer. It has a dual-polarized response and can achieve over 360° available transmission phase coverage. When used in meta-lens design, excellent radiation performances were achieved, with a maximum gain of 31.15 dBi at 28 GHz, a 3 dB gain bandwidth from 26.4 GHz to 30 GHz (12.86%) and an aperture efficiency of 42.7%. We believe that this ultrathin dual-polarized Huygens meta-lens has critical application in the antenna engineering field.

## 2. The EM Response of Huygens’ Unit

### 2.1. The EM Response of the Initial Huygens Unit

**Figure 1:**
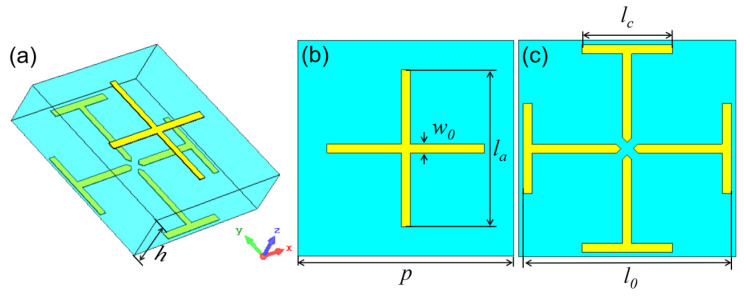
(**a**–**c**) Three-dimensional view, top view and bottom view of the unit.

Figure 1 shows the configuration of the initial Huygens unit. The Huygens unit has a double-layer metallic pattern on a single-layer dielectric substrate. The top pattern is a cross structure, and the bottom one is a center-split Jerusalem cross structure. The dielectric substrate is Rogers RO4003C with a dielectric constant of *ε_r_* = 3.55 and loss tangent of *tanδ* = 0.0027. It is a low-dielectric-constant substrate that is beneficial for improving the transmission performance and increasing the bandwidth. The thickness of the substrate is *h* = 1.27 mm. The period of the unit is *p* = 4.8 mm, and the width of the metallic wire is *w*_0_ = 0.2 mm. The varied structural parameters *l_0_, l_a_* and *l_c_* were used to tune the EM response of the unit. Due to the symmetry, the unit has a dual-polarized EM response. Thus, we only analyzed its *y*-polarized EM response. The EM responses of the unit were simulated using the commercial software CST Microwave Studio. 

**Figure 2:**
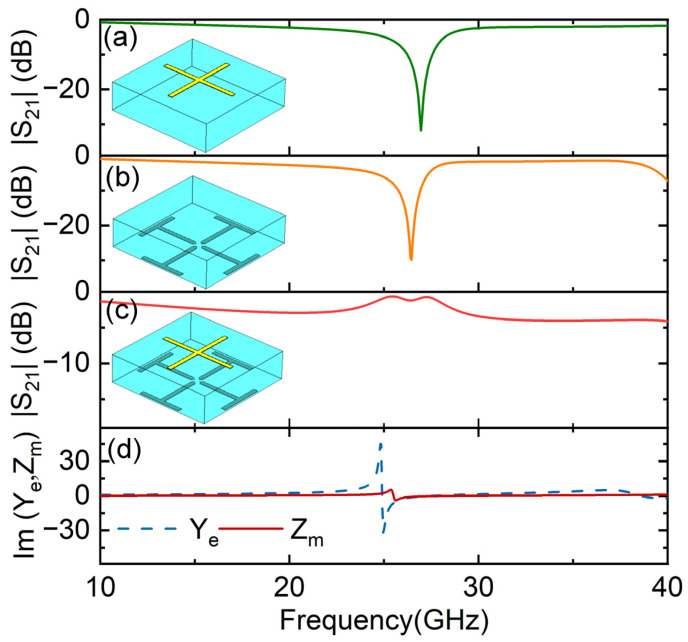
Transmission amplitude of (**a**) solo top element, (**b**) solo bottom element and (**c**) Huygens’ unit. Huygens’ resonance can be characterized by the imaginary part of the electric surface admittance Im[*Y_e_*] and magnetic surface impedance Im[*Z_m_*], which is shown in (**d**).

In order to observe Huygens’ resonance, we firstly considered the parameters *l*_0_ = 4.6 mm, *l_a_* = 3.5 mm and *l_c_* = 2 mm. When only considering the top pattern, a strong reflection appeared at 27 GHz, as shown in Figure 2a. A similar strong reflection phenomenon appeared at 26.4 GHz when only considering the bottom pattern, which is shown in Figure 2b. Obviously, such a strong reflection comes from the electric resonance of the frequency-selective surface pattern. When the top and bottom patterns were both present on the dielectric substrate, surprisingly, a transmission peak appeared at 25.2 GHz, with a transmission amplitude of −0.6 dB, as shown in Figure 2c. The EM response of the units can be characterized by the electric surface admittance and magnetic surface impedance, which are given by *Y_e_* = 2(1 − *T* − *R*)/(1 + *T* + *R*) and *Z_m_* = 2(1 − *T* + *R*)/(1 + *T* − *R*), where *R* is the reflection coefficient, and *T* is the transmission coefficient [15]. It can be seen from Figure 2d that both the electric surface admittance and magnetic surface impedance appeared around 25 GHz. The magnetic resonance is caused by odd-mode coupling between the top and bottom electric dipoles. Then, the balance between them stimulates Huygens’ resonance and thus induces resonant transmission. 

**Figure 3:**
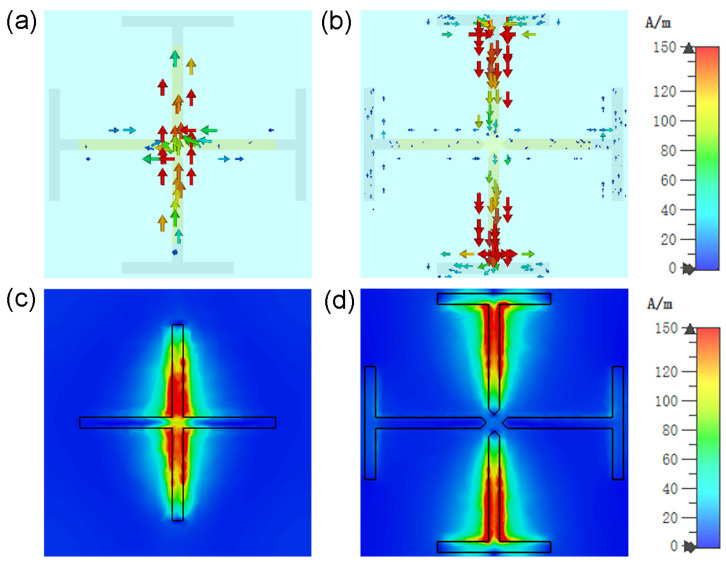
The distribution of the current and magnetic field on the unit at Huygens’ resonance. (**a**) the surface current on the top layer; (**b**) the surface current on the bottom layer; (**c**) the magnetic field near the top layer; (**d**) the magnetic field near the bottom layer.

The surface current distributions of the unit at a resonant frequency of 25.5 GHz are illustrated in Figure 3a,b. It can be seen that the currents flowed in opposite directions on the top and bottom surfaces. The currents with opposite directions formed a loop, thereby generating an orthogonal magnetic field. This was verified by the magnetic field distribution of the unit, which is shown in Figure 3c,d. 

**Figure 4:**
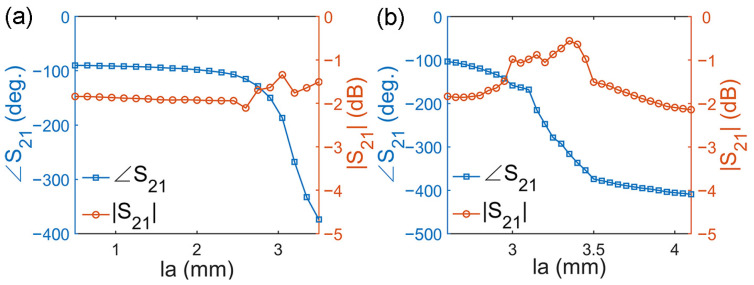
The transmission amplitude and phase of the unit (**a**) for scheme 1 and (**b**) for scheme 2.

The available transmission phase coverage can be obtained by varying *l*_0_, *l_a_* and *l_c_*. Here, we tested two schemes to obtain optimized results. For the first scheme, we fixed *l_c_* as 2 mm and set *l*_0_ to 4.6 − 0.1Δ mm and *l_a_* to 3.5 – 0.15Δ mm, where Δ, as an intermediate variable, increased from 0 to 20. For the second scheme, we fixed *l*_0_ as 4.6 mm and set *l_a_* to 3.5 – 0.05Δ mm and *l_c_* to 2 – 0.1Δ mm, where Δ, as an intermediate variable, increased from 0 to 9. In the first scheme, when *l_a_* changed from 0.5 mm to 3.5 mm, as shown in Figure 4a, the transmission amplitude was always greater than −2 dB, and the transmission phase shifted from −90° to −374°, covering a range of 284°. In the second scheme, when *l_a_* changed from 2.6 mm to 4.1 mm, as shown in Figure 4b, the transmission amplitude was always greater than −3 dB, and the transmission phase shifted from −103° to −409°, covering a range of 306°. The results show that, when combining these two schemes, 319° available transmission phase coverage can be obtained (from −90° and −409°). Moreover, the results also show that the second scheme achieved a greater available transmission phase coverage, but the first scheme had a better transmission amplitude. 

**Figure 5:**
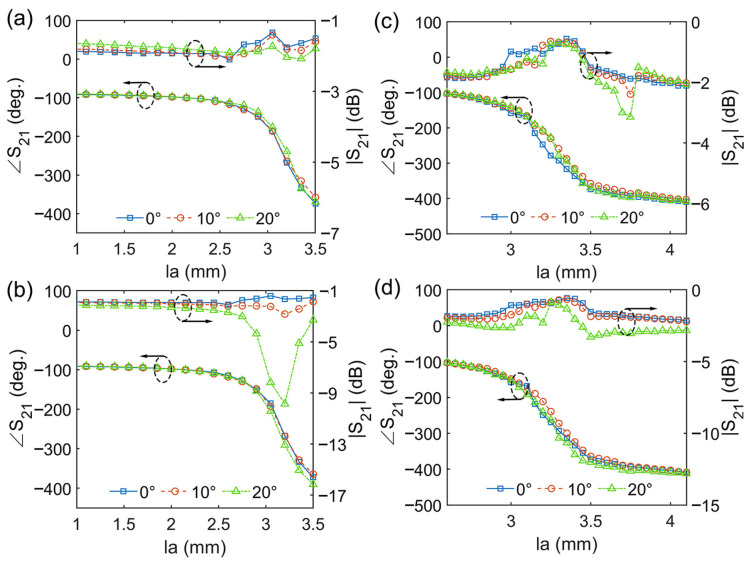
The angular responses of the unit for oblique incidence: (**a**) scheme 1 for TE oblique incidence; (**b**) scheme 1 for TM oblique incidence; (**c**) scheme 2 for TE oblique incidence; (**d**) scheme 2 for TM oblique incidence.

The above analysis concerns the normal incidence situation. In applications of planar lens antennas, it is necessary to investigate the case of oblique incidence. Cases of TE- and TM-polarized oblique incidence were considered, and the incident angle increased from 0° to 20°. The transmission amplitudes and phases of the unit under different incident angles are illustrated in Figure 5. It can be seen that, for both the TE and TM oblique incidences, when the incident angle increased from 0° to 20°, the transmission amplitude under most parameters was still greater than −3 dB. Moreover, the change in the transmission phase was relatively small. This shows that the angular stability of the EM response of the unit is good under incident angles ranging from 0° to 20°. On the other hand, we also noticed that for scheme 1, when the TM incidence angle reached 20°, the transmission amplitude under fewer parameters decreased sharply. It can be foreseen that as the incident angle further increases, there will be more cases of a sharp decrease in the transmission amplitude. This indicates that, when this unit is used for the manipulation of transmitted waves, the oblique angles of the EM waves cannot be too large. 

### 2.2. Optimization of Huygens’ Unit

**Figure 6:**
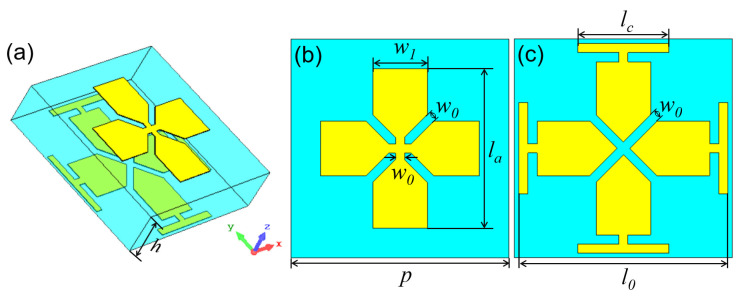
(**a**) Detailed structure of the optimized JC unit, (**b**) top view and (**c**) bottom view.

**Figure 7:**
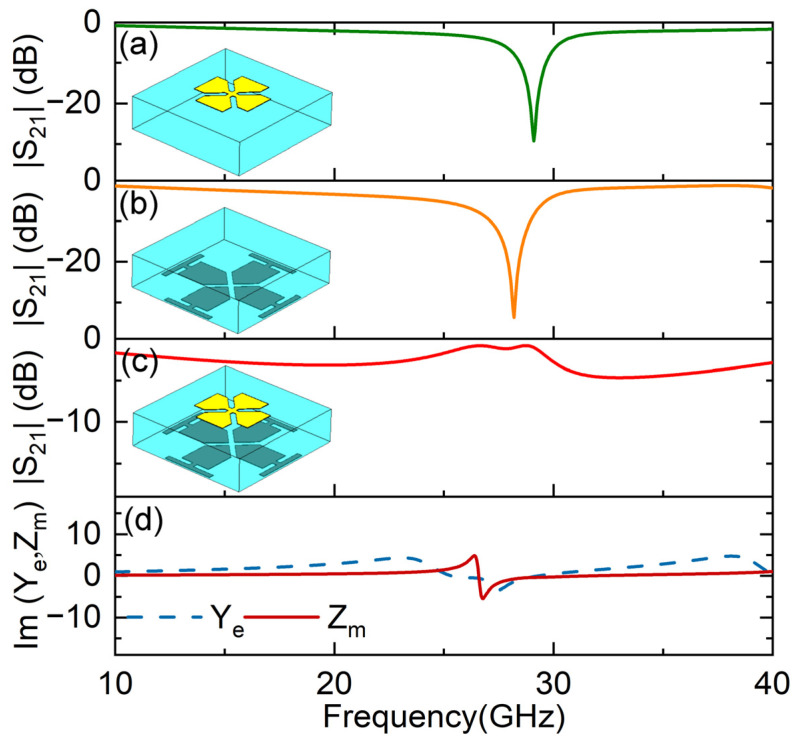
Transmission amplitude of (**a**) solo top pattern, (**b**) solo bottom pattern and (**c**) the optimized Huygens unit. Huygens’ resonance can be characterized by the imaginary part of the electric surface admittance Im[*Y_e_*] and magnetic surface impedance Im[*Z_m_*], which is shown in (**d**).

In order to further optimize the transmission amplitude and phase, we considered an optimized Huygens unit, as shown in Figure 6. The cross structure was replaced by a Jerusalem cross structure with width *w*_1_. The purpose of the optimization was to increase the overlapping area between the top and bottom patterns. Then, we considered the structural parameters *l*_0_ = 4.6 mm, *l_a_* = 2.4 mm, *l_c_* = 2 mm and *w*_1_ = 1.2 mm. The transmission spectra are presented in Figure 7. It can be seen in Figure 7a,b that, when considering the top and bottom patterns separately, they are both electric resonant structures, strongly reflecting the EM waves at a resonant frequency of 29 GHz and 28.2 GHz, respectively. However, when combining the top and bottom patterns, a resonant transmission peak appeared at 26.5 GHz, with a transmission amplitude of −0.86 dB, which is shown in Figure 7c. The electric and magnetic resonances appeared around 26.5 GHz, as shown in Figure 7d.

**Figure 8:**
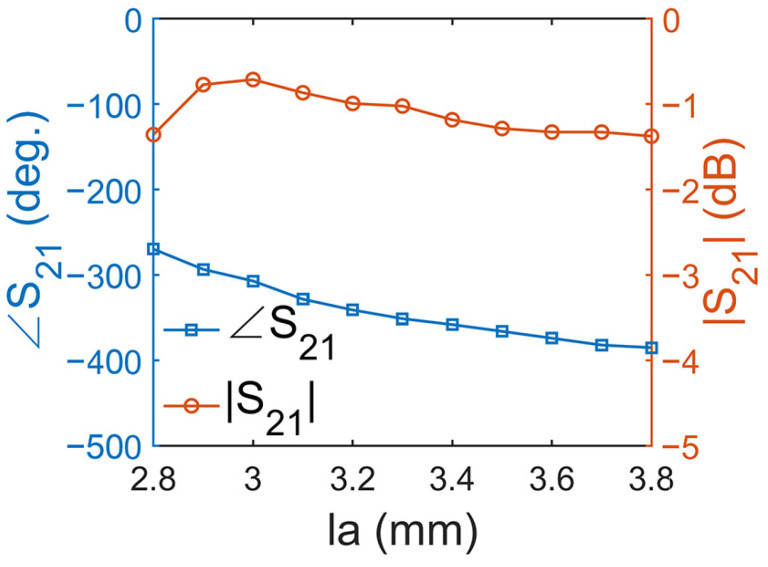
The phase and amplitude of the unit with varied *l_a_* and *w_1_*.

Then, we considered a third scheme to achieve optimized transmission amplitudes and phase responses. In this scheme, we fixed the parameter *l*_0_ as 4.6 mm and set *l_a_* to 3.5 − 0.1Δ mm and *w*_1_ to 0.2 + 0.05Δ mm, where Δ, as an intermediate variable, increased from 0 to 10. It can be seen in Figure 8 that, when *l_a_* changed from 2.8 mm to 3.8 mm, the transmission phase shifted from −269° to −385°, and the transmission amplitude was optimized, reaching above −1.4 dB. By combining these three schemes, approximately 317° available transmission phase coverage can be obtained, with a transmission amplitude greater than −1.72 dB. Some representative structural parameters and relative transmission amplitudes and phase responses are listed in Table 1. 

## 3. Application of Huygens’ Unit in Meta-Lens

**Figure 9:**
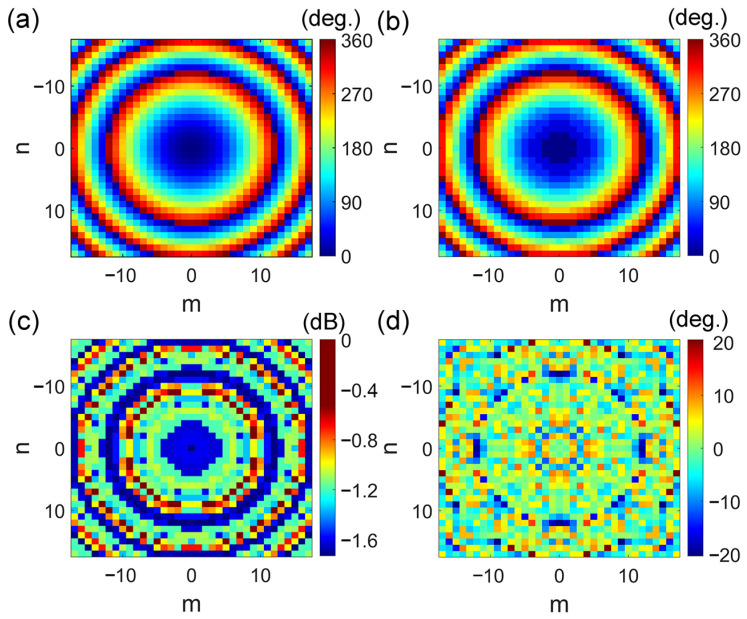
(**a**) Theoretical phase distribution, (**b**) actual phase distribution, (**c**) transmission amplitude for phase compensation and (**d**) the error between the theoretical and actual phase distribution.

**Figure 10:**
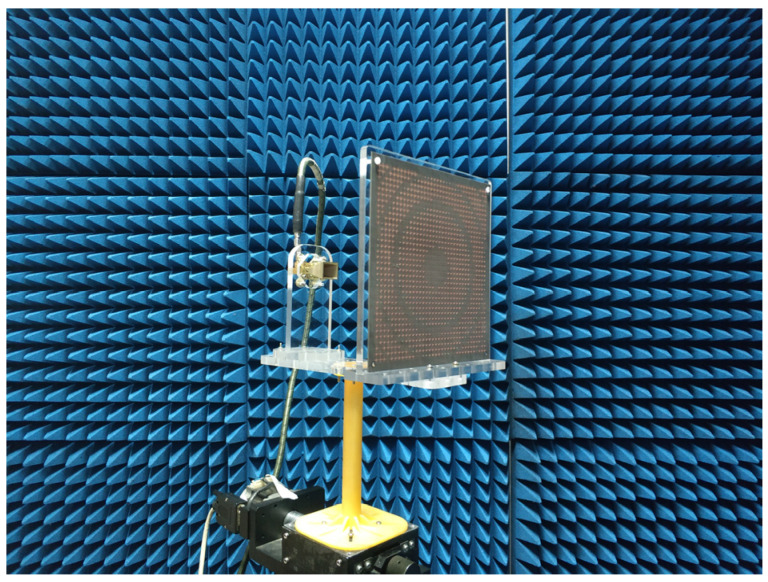
The fabricated Huygens meta-lens antenna and its test environment setup.

The Huygens unit can be used for the design of an ultrathin dual-polarized meta-lens. Considering a 15 dBi standard-gain horn antenna as a feeding source vertically placed on the meta-lens with a distance between them of *F* (i.e., focal length), the phase distribution Δ*φ*(*m*, *n*) on the meta-lens can be calculated using the following formula:

(1) $$\Delta \varphi (m,n)=\frac{2\pi }{\lambda_{0}}(\sqrt{{\left(mp\right)}^{2}+{\left(np\right)}^{2}+F^{2}}-F)$$

where *λ*_0_ is the working wavelength, and Δ*φ*(*m*, *n*) is the phase difference between the unit cell at (*m*, *n*) on the surface and that at the origin (0, 0). A working wavelength of λ_0_ = 10.7 mm was chosen (corresponding to a frequency of 28 GHz). The focal length was *F* = 159 mm, and the size of the meta-lens was 168 × 168 mm^2^ (35 × 35 units). The focus-to-diameter ratio (F/D) was 0.95, corresponding to a maximum oblique incident angle of 27.8°. This angle is acceptable since the radiation of the horn antenna at the edge of the meta-lens is much weaker than that at the center. The calculated and actual phase distributions of the meta-lens are illustrated in Figure 9a,b. The phase error between the design and the theoretical calculation was very small, as shown Figure 9d. Thereby, the design is consistent with the theoretical calculation. The transmission amplitude distribution on the meta-lens exhibited a good transmission performance, as shown in Figure 9c. Then, the meta-lens was fabricated according to the calculated phase distribution. The meta-lens prototype was assembled on a rotating platform in a microwave anechoic chamber to measure its far-field radiation performance, including co-polarization and cross-polarization, which is illustrated in Figure 10.

**Figure 11:**
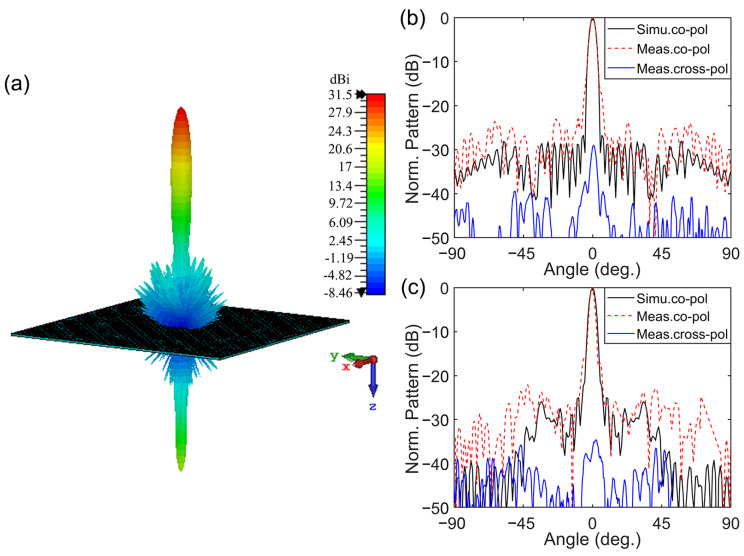
Far-field radiation patterns of the meta-lens at 28 GHz. (**a**) Three-dimensional pattern, and (**b**,**c**) two-dimensional normalized patterns for the x- and y-polarizations, respectively.

**Figure 12:**
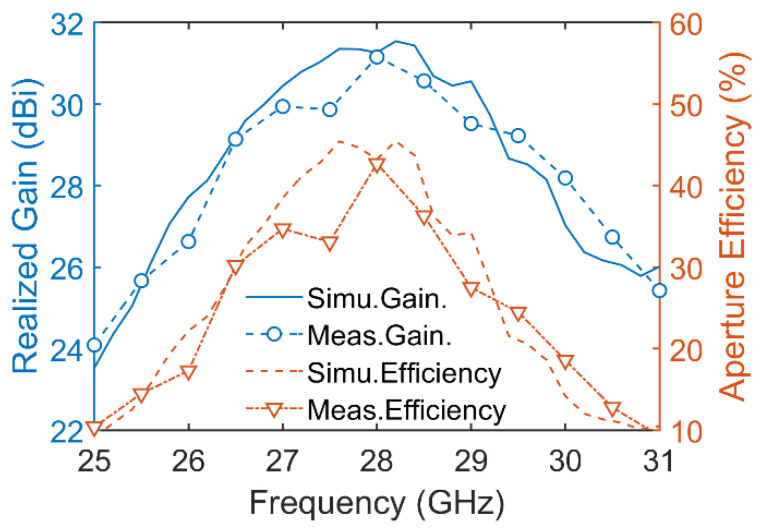
Simulated and measured realized gain spectrum and aperture efficiency of the meta-lens.

The simulated and measured far-field radiation patterns of the meta-lens at 28 GHz are shown in Figure 11. The simulated 3D radiation pattern in Figure 11a shows that a high-directional transmitted beam was generated with a gain of 31.5 dBi at 28 GHz. The sidelobe and backlobe levels were −28 dB and −14 dB, respectively. The measured results in Figure 11b,c show that the x- and y-polarized sidelobe levels were −26.8 dB and −26.51 dB, respectively. These values are in agreement with the simulated results. Moreover, the cross-polarization levels for both polarizations were 30 dB lower than the co-polarization levels. The gain spectra of the meta-lens are illustrated in Figure 12. The simulated results show that the maximum gain was 31.5 dBi at 28.2 GHz, with an aperture efficiency of 45.7%, and that the 3 dB gain bandwidth ranged from 26.3 GHz to 29.5 GHz, or 11.42%. The measured results show that the maximum gain was 31.15 dBi at 28 GHz, with an aperture efficiency of 42.7%, and that the 3 dB gain bandwidth ranged from 26.4 GHz to 30 GHz, or 12.86%. The simulated and measured results consistently demonstrate the excellent radiation performance of the meta-lens antenna.

The important performance indicators of the proposed meta-lens antenna are compared with those of state-of-the-art planar lens antennas in Table 2. Compared with the multilayer FSS [28,29] and double-layer design with vias [34], our design has the merits of being easy to fabricate and having a low assembly cost. On the one hand, the bandwidth of our design is slightly smaller than that of the multilayer FSS design, but it entirely covers the 28 GHz band (26.5–29.5 GHz). In addition, both this work and [31] were designed based on the induced magnetic mechanism and achieved similar performances, but they studied different structural topologies. In the future, we expect to combine these two works to achieve dual-band, dual-polarized double-layer meta-lens antennas, which have important applications in millimeter-wave communication systems. 

## 4. Conclusions

In this paper, a double-layer Huygens unit is proposed, which is composed of a cross pattern and a center-split Jerusalem cross pattern on both sides of a dielectric substrate. By optimizing the unit, an available phase coverage of nearly 360° with a transmission amplitude greater than −1.72 dB was achieved. Such transmitted EM responses of Huygens’ unit can be used for the design of double-layer dual-polarized meta-lenses. The merits of this design include its very simple structure, making it easy to fabricate. Additionally, its disadvantages are that the bandwidth is not wide enough and can only be used for a single band. In the future, we expect to further increase its bandwidth and expand its function to dual bands. We believe that such a design helps to promote the important applications of Huygens’ meta-lens in millimeter-wave communication systems. 

## Figures and Tables

**Table 1:** Some representative structural parameters and relative transmission amplitude and phase.

*l*_0_ (mm)	*l_a_* (mm)	*l_c_* (mm)	*w*_1_ (mm)	Transmission Amplitude (dB)	Phase Shift (Degree)
1.6	1.1	0.5	1.15	−1.66	−86
3	1.52	1.52	1.32	−1.68	−96
3.6	1.82	1.7	1.3	−1.56	−106
4	1.92	1.76	1.25	−0.99	−127
4.2	2	1.8	1.3	−0.75	−138
4.25	2.05	1.82	1.2	−0.88	−140
4.35	2.1	1.85	1.2	−0.83	−150
4.46	2.12	1.85	1.2	−1.2	−163
4.45	2.19	1.89	1.2	−0.7	−176
4.45	2.25	1.89	1.2	−0.53	−193
4.45	2.3	1.89	1.2	−1	−208
4.55	2.33	1.97	1.2	−1.1	−228
4.6	2.35	1.95	1.24	−1.35	−248
4.6	2.4	2	1.2	−1.14	−256
4.6	2.45	2	1.2	−1.6	−271
4.6	2.5	2.05	1.15	−1.2	−282
4.6	2.6	2.1	1.1	−1.08	−305
4.6	2.68	2.15	1.08	−1.05	−319
4.6	2.78	2.18	1.03	−1.18	−332
4.6	2.87	2.25	1	−1.19	−347
4.6	3.5	2	0.2	−1.63	−371
4.6	4.1	2.08	0.2	−1.6	−399
4.6	4.12	2.12	0.2	−1.72	−403

**Table 2:** Comparison of planar lens antennas.

Ref	Layer Number and Style	Frequency (GHz)	*F*/*D* Ratio	Max. Gain (dBi)	Bandwidth (%)	Aperture Efficiency (%)
[28]	4-FSS	13.5	0.95	30.22	9.8 *	50.0
[29]	4-FSS	11.7	0.81	33.8	12.6 *	51.2
[34]	2-FSS with vias	20.0	1.24	33.0	5.9 *	40.0
[31]	2-Huygens	28.0	0.95	31.6	14.1 **	50.0
This work	2-Huygens	28.0	0.95	31.5	12.86 **	42.7

* 1 dB bandwidth; ** 3 dB bandwidth.

## Data Availability

Data will be made available on request.
